# Trichosanthin cooperates with Granzyme B to restrain tumor formation in tongue squamous cell carcinoma

**DOI:** 10.1186/s12906-021-03266-6

**Published:** 2021-03-09

**Authors:** Zeyao Zhu, Zhenguang Ying, Meiqi Zeng, Qiang Zhang, Guiqing Liao, Yunliu Liang, Chunman Li, Chengfei Zhang, Xia Wang, Weipeng Jiang, Ping Luan, Ou Sha

**Affiliations:** 1 School of Dentistry, Shenzhen University Health Science Center, Shenzhen, China; 2 School of Basic Medical Sciences, Shenzhen University Health Science Center, Shenzhen, China; 3 The Shenzhen Stomatology Hospital, Shenzhen, China; 4 Department of Oral and Maxillofacial Surgery, Guanghua School of Stomatology, Sun Yat-Sen University, Guangzhou, China; 5 Faculty of Dentistry, University of Hong Kong, Hong Kong, China

**Keywords:** Apoptosis, Granzyme B (GrzB), Oral squamous cell cancer (OSCC), Tongue squamous cell cancer (TSCC), Trichosanthin (TCS)

## Abstract

**Background:**

Tongue squamous cell carcinoma (TSCC) is a common type of oral cancer, with a relatively poor prognosis and low post-treatment survival rate. Various strategies and novel drugs to treat TSCC are emerging and under investigation. Trichosanthin (TCS), extracted from the root tubers of Tian-Hua-Fen, has been found to have multiple biological and pharmacological functions, including inhibiting the growth of cancer cells. Granzyme B (GrzB) is a common toxic protein secreted by natural killer cells and cytotoxic T cells. Our group has reported that TCS combined with GrzB might be a superior approach to inhibit liver tumor progression, but data relating to the use of this combination to treat TSCC remain limited. The aim of this study was to examine the effectiveness of TCS on TSCC processes and underlying mechanisms.

**Methods:**

First, we screened the potential antitumor activity of TCS using two types of SCC cell lines. Subsequently, a subcutaneous squamous cell carcinoma xenograft model in nude mice was established. These model mice were randomly divided into four groups and treated as follows: control group, TCS treatment group, GrzB treatment group, and TCS/GrzB combination treatment group. Various tumorigenesis parameters, such as Ki67, PCNA, caspase-3, Bcl-2 and VEGFA, et al., were performed to determine the effects of these treatments on tumor development.

**Results:**

Screening confirmed that the SCC25 line exhibited greater sensitivity than the SCC15 line to TCS in vitro studies. TCS or GrzB treatment significantly inhibited tumor growth compared with the inhibition seen in the control group. The TCS/GrzB combination inhibited tumor growth more than either drug alone. TCS treatment inhibited tumor proliferation by downregulating Ki67 and Bcl2 protein expression while accelerating tumor apoptosis. In the TCS/GrzB-treated group, expression of Ki67 was further downregulated, while the level of activated caspase-3 was increased, compared with their expression in either of the single drug treatment groups.

**Conclusion:**

These results suggest that the TCS/GrzB combination could represent an effective immunotherapy for TSCC.

## Background

Oral squamous cell carcinoma (OSCC) is the most common oral cancer; it is characterized by a high degree of local invasiveness and proliferation capacity and a relatively poor prognosis [1–3]. Among OSCCs, tongue squamous cell cancer (TSCC) has the highest incidence and is often associated with a low survival rate [4, 5]. TSCC also results in the highest rates of mortality among head and neck region cancers [6, 7]. Thus, it is imperative to explore novel agents or treatment strategies targeting TSCC.

Tian-Hua-Fen (*Trichosanthes kirilowii* Maxim.) is a plant renowned in traditional Chinese medicine and is used as an abortifacient based on its directed toxicity toward trophoblasts and choriocarcinoma cells [8–11]. Trichosanthin (TCS), extracted from the root tubers of Tian-Hua-Fen, is a type 1 ribosome-inactivating protein (RIP). It has been reported that treating tumor cells with TCS causes cell death by inducing cell necrosis and inhibiting cellular protein synthesis [12]. Recently, a series of studies have revealed the antitumor effects of TCS, suggesting it has apoptotic activities in numerous types of tumors, including breast cancer, nasopharyngeal carcinoma, hepatocellular carcinoma, non-small cell lung cancer, cervical cancer, and B-cell lymphoma [11, 13–18]. Therefore, TCS represents a potential novel therapeutic drug for antitumor treatment.

As a major constituent involved in cytotoxic T lymphocyte (CTL)-mediated tumor cell apoptosis, the mechanism of granzyme B (GrzB)-mediated cell death has been reasonably well defined [19, 20]. In a previous study, our group reported that TCS increases GrzB penetration of tumor cells by upregulating the cation-independent mannose-6-phosphate receptor (CI-MPR) [17]. These observations suggested that TCS combined with GrzB might be a superior approach to enhance the efficacy of cancer immunotherapy [17]. Nevertheless, the antitumor effects of TCS and GrzB in TSCC remain largely unknown. The present study aimed to evaluate the potential antitumor activity of TCS or GrzB alone versus TCS and GrzB combined. Further studies were also conducted to explore the molecular mechanisms regulating TCS and/or GrzB targeting TSCC.

## Methods

### Cell culture

Cells of the human squamous cell carcinoma lines SCC15 and SCC25 were purchased from the American Type Culture Collection and cytogenetically tested and authenticated prior to freezing. The two cell lines were routinely passaged in a 1:1 mixture of Dulbecco’s modified Eagle’s medium and Ham’s F12 medium (Gibco, Thermo) containing 1.2 g/L sodium bicarbonate, 2.5 mM L-glutamine, 15 mM HEPES, and 0.5 mM sodium pyruvate supplemented with 400 ng/mL hydrocortisone, 90% fetal bovine serum (Gibco, Thermo), 10% 100 U/mL penicillin, and 100 μg/mL streptomycin at 37 °C in a 5% CO_2_ incubator.

### Xenograft tumor model

All animal experimental procedures were approved by the Institutional Animal Care and Use Committee (IACUC) of Peking University Shenzhen Graduate School. Eight-week-old male BALB/c nude mice (Guangdong Medical Laboratory Animal Center, Guangzhou) were maintained under specific pathogen-free (SPF) conditions and were free to access sterilized food pellets and distilled water, with a 12-h light/dark cycle. Each mouse was subcutaneously inoculated with 2 × 10^6^ SCC25 cells in the right hind-limb. Three days later, tumor formation was assessed in each mouse for further drug treatment.

### Trichosanthin and granzyme B treatment

The SCC25 tumor-bearing mice were randomly divided into four groups: phosphate-buffered saline (PBS) control group, TCS group, GrzB group, and TCS/GrzB combination group (*n* = 8–9 per group). TCS protein was extracted in our laboratory, while recombinant granzyme B (active) was purchased from Sino Biological. TCS (2 μg/g body weight) was diluted in 60 μL PBS and intraperitoneally injected on days 1, 3, 5, and 7 in the TCS and combination groups. Active GrzB (2 μg/100 g body weight) was diluted in 60 μL PBS and intraperitoneally injected on days 2, 4, 6, and 8 in the GrzB and combination groups. Mice in the PBS group were intraperitoneally injected with 60 μl PBS each day during the administration period. Tumors were assessed every other day using Vernier calipers, and tumor volumes were calculated using the elliptical formula: 1/2 (long diameter × short diameter^2^). On day 16, all mice were sacrificed by cervical dislocation, and tumors were weighed after being separated from the surrounding muscle and dermis. For each group, tumor tissues were collected and divided into three samples: the first sample was homogenized into tumor lysis for western blot analysis, the second sample was fixed with 10% neutral formalin and embedded in paraffin for hematoxylin and eosin (HE) staining and immunofluorescence analysis, and the third sample was stored in liquid nitrogen for RNA extraction. The tumor inhibition rate was calculated using the following equation: Tumor inhibition rate (%) = (1 − average volume of experimental group / average volume of control group) × 100%.

### Hematoxylin and eosin staining and morphological analysis

The fresh tumor tissues were washed with normal saline then placed in 10% neutral formalin solution and stored overnight at 4 °C. The next day, the fixed samples were dehydrated, embedded in paraffin, and sectioned at 6-μm thickness using a Leica microtome. After drying overnight, the slides were stained with H&E according to the standard protocol [21]. The stained sections were dehydrated with ethanol, rendered transparent with xylene, and sealed. The tissue morphology profiles were observed under an optical microscope (Olympus, Japan).

### Western blot analysis

Total protein was extracted by lysing fresh tumor tissues in precooling RIPA buffer (Thermo), supplemented with protease inhibitor cocktail (MCE) and PhosSTOP™ phosphatase inhibitor (Roche), quantitated using a Pierce™ BCA Protein Assay (Thermo) and transferred onto a PVDF membrane (Millipore) after separation by SDS-PAGE. The membranes were incubated with respective primary antibodies at 4 °C overnight then incubated with horseradish peroxidase (HRP)-conjugated secondary antibody, as previously described [17]. Chemiluminescence was developed using ECL Ultra HRP substrate (Merck) and photographed under the SAGECREATION ChemiMini™ Imaging System.

### Quantitative real-time PCR

Total RNA was isolated from tumor tissues using the Eastep™ Super Total RNA Extraction Kit (Promega) and reverse transcribed into cDNA using the PrimeScript™ RT Reagent kit (TaKaRa), according to the manufacturer’s instructions. The synthesized cDNA was then amplified by quantitative PCR using TB Green Premix Ex Taq (TaKaRa) on a Quantstudio™ 7 Flex Real-Time PCR System (ABI). The expression of target genes was normalized against GAPDH using the 2^-ΔΔCt^ assay. Primer oligos were synthesized by TSINGKE Biological Co., Ltd., (Beijing, China) and are listed in Table 1.

**Table 1:** Primers

P53	P53-qPCR-F	TAGTGTGGTGGTGCCCTATG
	P53-qPCR-R	CCAGTGTGATGATGGTGAGG
BAD	Bad-qPCR-F	TACCTGCCTCTGCCTTCCA
	Bad-qPCR-R	CTGCTCACTCGGCTCAAACT
VEGFA	VEGFA-qPCR-F	AGGGCAGAATCATCACGAAGT
	VEGFA-qPCR-R	AGGGTCTCGATTGGATGGCA
GAPDH	gapdh-qPCR-F	GTCAACGGATTTGGTCGTATTG
	gapdh-qPCR-R	CATGGGTGGAATCATATTGGAA

### Histology and immunofluorescence

The paraffin-embedded tissue sections (6 μm) were deparaffinized by immersion in fresh xylene and hydrated by immersion in graded ethanol washes. The sections were then immersed in citrate buffer (pH 6.0) at 95 °C for 20 min for antigen retrieval before being incubated with 3% donkey serum at room temperature for 30 min. The slides were then incubated with one of the following primary antibodies: monoclonal rabbit anti-human cleaved caspase-3 (clone 5A1E, Cell Signaling Tech.) at 1:100; monoclonal rabbit anti-human PCNA (clone D3H8P, Cell Signaling Tech.) at 1:100; monoclonal rabbit anti-human Ki67 (Cell Signaling Tech.) at 1:2000; monoclonal rabbit anti-human caspase-7 (Cell Signaling Tech.) at 1:200, or monoclonal mouse anti-human Bcl2 (sc-7382, Santa Cruz) at 1:100 overnight at 4 °C in a humidified box. The next day, after washing in PBST, the sections were incubated with Alexa Fluor 488 or Alexa Fluor 555 goat anti-mouse secondary Ab (Thermo) (1:200 dilution) for 2 h at room temperature in a humidified box in the dark. Next, nuclear staining was performed with 4′,6-diamidino-2-phenylindole (DAPI) (Cell Signaling Tech.). Finally, the fluorescence was observed and images were taken under a FV3000 confocal microscope (OLYMPUS, Japan).

### TUNEL staining assay

For the in situ terminal deoxynucleotidyl transferase-mediated dUTP nick-end labeling (TUNEL) assay, procedures were performed using the DeadEnd™ Fluorometric TUNEL System (Promega, USA). After deparaffinizing and hydrating, the staining procedures were carried out according to the manufacturer’s instructions. Sections were stained with DAPI and mounted using ProLong® Gold Antifade Reagent (Cell Signaling Tech.). The nuclei-stained green was an apoptotic cell. The Bcl-2/Bcl-xL inhibitor, ABT-263 (8 μM; Selleckchem, Houston, TX, USA), was used as a positive control. Apoptosis was inhibited using Z-VAD-FMK (pan-caspase inhibitor). Three fields were chosen at random and analyzed for positive signaling. The apoptotic index was calculated as the percentage of positive-stained cells: number of apoptotic cells (green)/total number of nucleated cells.

### Statistical analysis

Data (mean ± SEM, 2–3 experiments) were analyzed for statistical significance using a *t*-test. A *p*-value < 0.05 was considered statistically significant. GraphPad Prism (GraphPad Software, San Diego, CA, USA) was used to perform the statistical analyses.

## Results

### TCS reduces the viability of OSCC cell-line cells

**Fig. 1:**
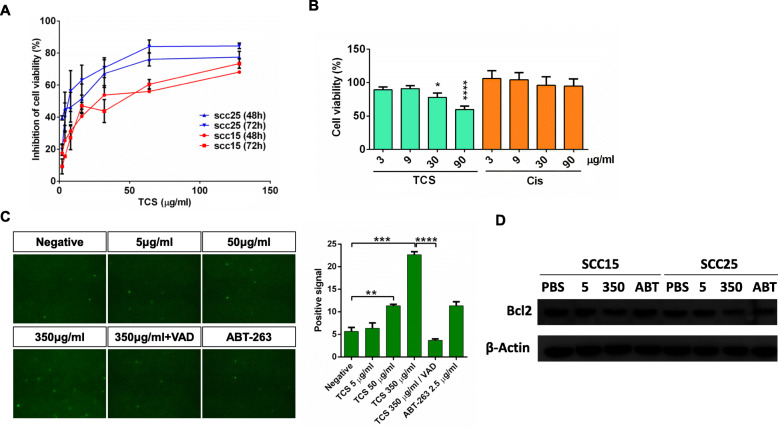
Effect of TCS on the viability of oral cancer cells in vitro. **a**. SCC15 and SCC25 cells were cultured in the presence of different concentrations of TCS for 48 h to 72 h. The remaining cell viability was measured by CCK8 assay. **b**. Cell viability of SCC-25 cells upon treatments of TCS and cisplatin for 72 h at the indicated concentrations. Cell viability was evaluated by MTS assay. Only TCS at higher concentrations displayed statistically significant reductions relative to the lower group (*n* = 5). **c**. TCS induced the apoptosis of SCC25 cells in a concentration-dependent manner. TUNEL positive signal cells were captured following treatment with TCS (5, 50, and 350 μg/mL) for 48 h. Z-VAD-FMK (pan-caspase inhibitor, 20 μM) treatment inhibited TCS-induced for 48 h. ABT-263(8 μM) for 48 h was used as the positive control. (TUNEL: green). **d**. Western blotting analysis to detect Bcl-2 in SCC25 cells after various TCS treatments. Values are presented as the mean ± standard error of the mean from a minimum of three independent experiments. **, *p* < 0.01; ***, *p* < 0.001; ****, *p* < 0.0001

To investigate the potential antitumor effect of TCS on different types of oral cancer cells, the SCC15 and SCC25 squamous cell carcinoma cell lines were selected. The two cell lines were separately treated with different concentrations of TCS (ranging from 2 to 128 μg/mL) for 48 and 72 h. Then, cell viability was determined using a CCK-8 assay kit (Fig. 1 a). The SCC25 cell line exhibited a higher sensitivity to TCS compared with the sensitivity of the SCC15 cell line after treatment with TCS for 48 h, with an IC_50_ of 8.4 and 49 μg/mL, respectively. The SCC25 cell line also exhibited the greater sensitivity to TCS compared with the sensitivity of SCC15 after treatment with TCS for 72 h, with an IC_50_ of 9.2 and 34.7 μg/mL, respectively. The inhibiting effects of TCS and the traditional chemotherapeutic agent Cisplatin (Cis) on SCC-25 cells were also evaluated using an MTS assay. As shown in Fig. 1 b, increasing the concentration of TCS led to a significant decrease in cell viability; this was not observed upon increasing the concentration of Cis. Compared with Cis, TCS resulted in a significantly decreased viability at a lower concentration of 30 μg/ml. Therefore, SCC-25 cells appear to be more sensitive to TCS than to Cis. Based on these results (Fig. 1, a), SCC25 cells were chosen for further study.

### TCS induces apoptosis in OSCC cell lines

To assess whether apoptosis occurred in SCC25 cell lines treated with TCS, a TUNEL assay was applied to detect DNA fragmentation. The results showed that many more SCC25 cells underwent DNA fragmentation following treatment with TCS. TCS induced apoptosis of SCC25 cells in a concentration-dependent manner following treatment with TCS (5, 50, and 350 μg/mL) for 48 h (Fig. 1, b, c). SCC25 cells were treated with 350 μg/mL TCS combined with 20 μM Z-VAD-FMK for 48 h, and the Z-VAD-FMK treatment was shown to markedly inhibit TCS-induced apoptosis, suggesting that TCS-induced tumor cell death involved caspase activation (*P* < 0.0001; Fig. 1, c). Immunoblot analysis revealed that TCS treatment inhibited Bcl2 expression in a dose-dependent manner. In particular, a dosage of 350 μg/mL TCS significantly decreased the expression of Bcl2 in the two SCC cell lines (Fig. 1, d).

### TCS increases granzyme B to inhibit tumor growth

**Fig. 2:**
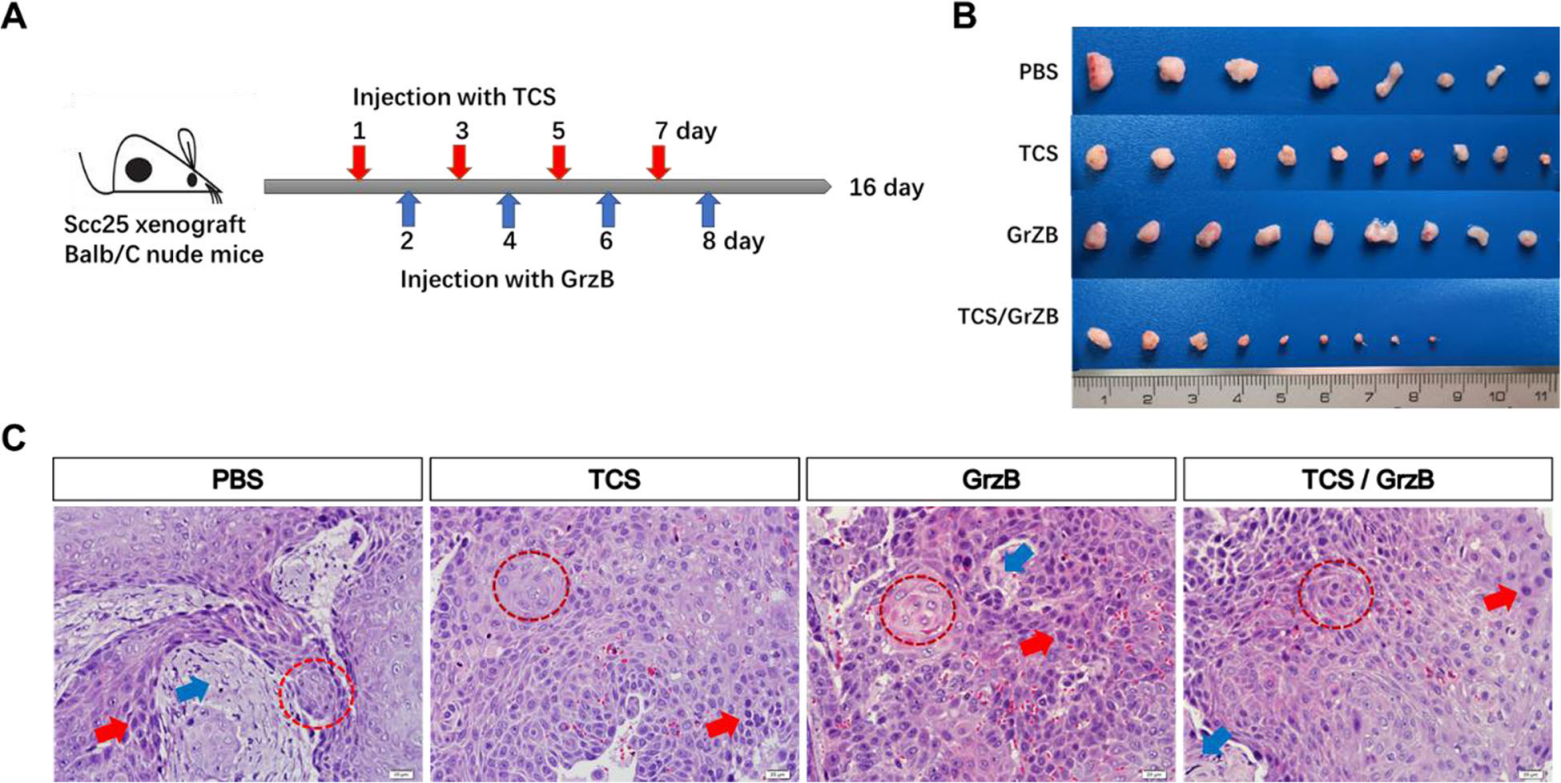
Establishment of SCC25 xenograft tumor model and treatment schedule in nude mice. **a**. Mice were administered TCS or GrzB through intraperitoneal injection according to the treatment schedule. **b**. On day 16, mice were sacrificed and excised tumors are shown in the right panel. **c**. HE staining results showed the typical tong tumor characteristics with a high nuclear/cytoplasmic ratio and a nest-like distribution in all of the groups. Tumor and stromal areas are indicated with red arrow and blue arrow, respectively

**Fig. 3:**
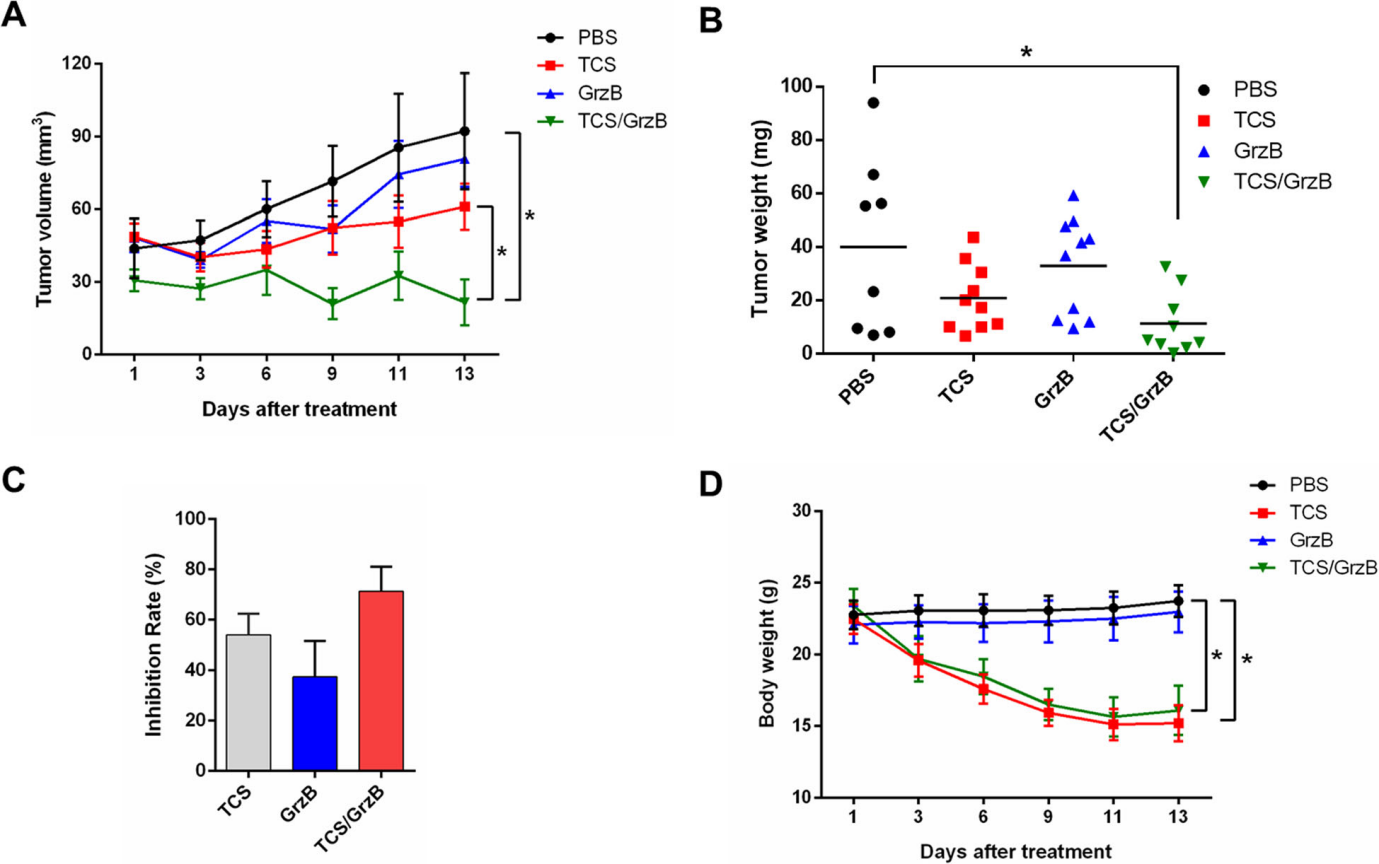
TCS and GrzB inhibits SCC25 xenograft tumor growth. **a**-**c**. The tumor size, weight and volume was significantly reduced by the treatment of TCS/GrzB. **d**. TCS combined with GrzB resulted in a more pronounced inhibition of tumor growth compared with either drug alone in the xenograft tumor model. G. Mouse body weight changed between the control and TCS-treated groups. Data are expressed as mean ± standard error of the mean. *, *p* < 0.05

To investigate the therapeutic effects of TCS and GrzB on TSCC in vivo, the SCC25 cell line was selected to establish a TSCC xenograft nude mouse model. Three days after injecting tumor cells, the mice were administered TCS and/or GrzB via intraperitoneal injection, according to the treatment schedule (Fig. 2, a). Tumor volumes were measured every other day. Mice were euthanized and tumors were excised and collected on day 16 (Fig. 2, b). Similar to other studies [22], typical tongue tumor characteristics were observed, with a high nuclear/cytoplasmic ratio (red arrow) and a nest-like distribution (red circle) in the tumor tissues (Fig. 2, c). Well-differentiated, typical keratin pearls (red circle) and tumor stroma areas (blue circle) were also found in all four treatment groups (Fig. 2, c). However, HE staining failed to reveal any morphological differences in tumor tissues among the four groups. Compared with the tumor growth in the PBS control group, TCS or GrzB treatment significantly suppressed tumor growth, as indicated by the reduced tumor weight and volume (Fig. 3, a and b). Treatment with a combination of TCS and GrzB led to more pronounced inhibition of tumor growth compared with the inhibition produced by either drug alone in the xenograft tumor model. TCS or GrzB treatment inhibited tumor growth by more than 20%, while TCS and GrzB combined inhibited tumor growth by up to 71.38% (Fig. 3, c). However, drug toxicities were also observed, and the body weight of mice in the treatment groups decreased (Fig. 3, d). Collectively, however, these results suggest that trichosanthin and granzyme B combination treatment was superior to either drug alone for inhibiting SCC25 tumor growth in vivo.

### A combination of TCS and GrzB inhibits tumor cell proliferation and induces apoptosis

**Fig. 4:**
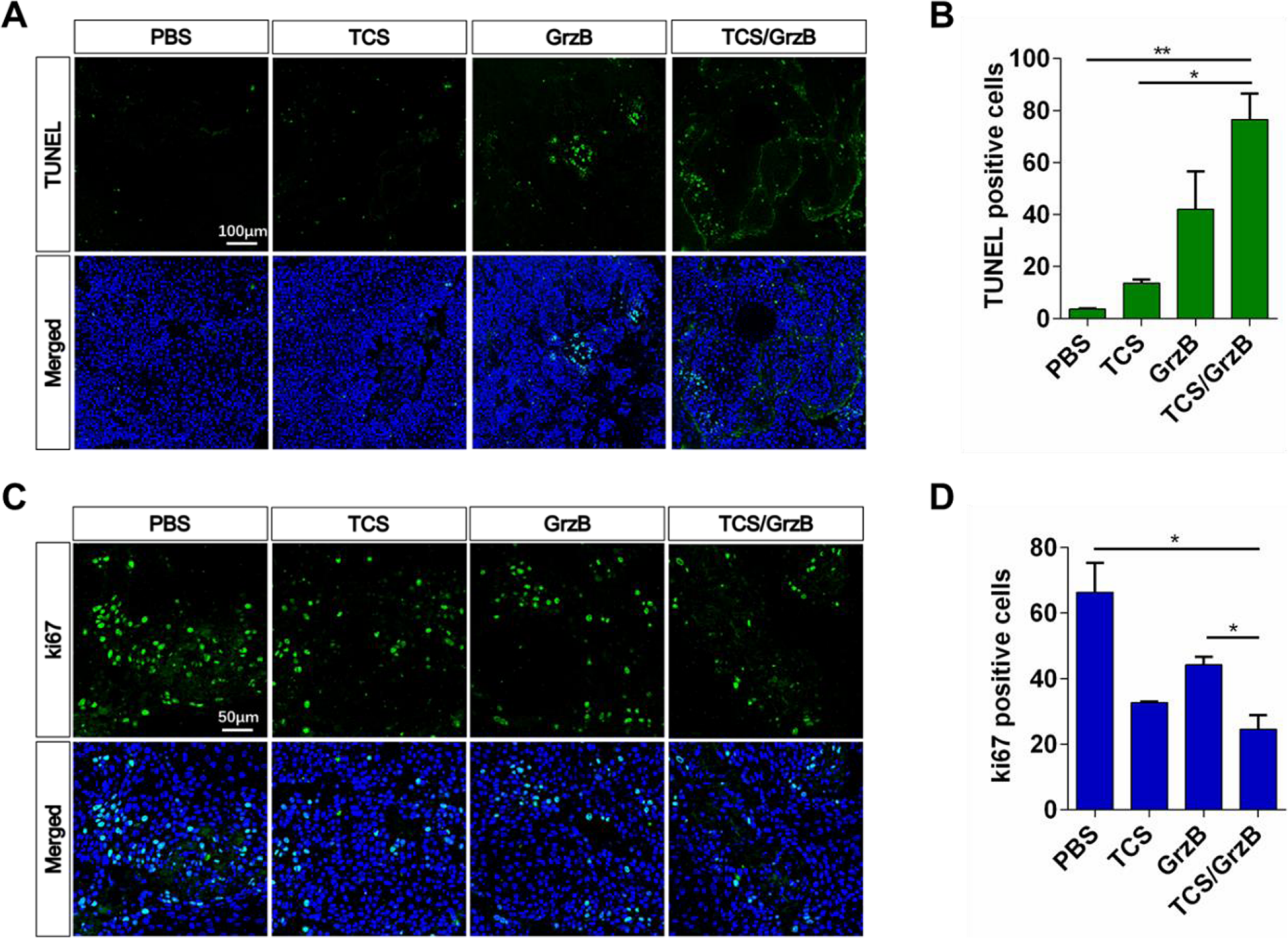
TCS and GrzB inhibits tumor cell proliferation and induces apoptosis. **a**, **b**. TUNEL positive signals were significantly increased by the treatment of GrzB and TCS promoted the apoptotic cell death of tumor cells (TUNEL: green; DAPI: blue). Bar = 100 μm **c**, **d**. Ki67 positive signals were significantly decreased by the treatment of GrzB and TCS inhibited the proliferation of tumor cells (Ki67: green; DAPI: blue). Data are expressed as mean ± standard error of the mean. Bar = 50 μm. *, *p* < 0.05; **, *p* < 0.01

To further elucidate the apoptotic mechanisms induced by TCS and GrzB in vivo, DNA fragmentation, a marker of apoptotic cells, was assessed using a TUNEL assay. Treatment with TCS and GrzB combined resulted in a higher number of apoptotic nuclei than the PBS control group or the other treatment groups (Fig. 4, a, b). Ki67, a typical marker of tumor cell proliferation [23], was assessed using anti-Ki67 antibody to reveal the effects of TCS and GrzB on tumor cell proliferation. It was shown that TCS and GrzB combination treatment significantly inhibited cell proliferation in tumor tissues compared with cell proliferation in the PBS control group (Fig. 4 c, d). Overall, these data suggest that treatment with a combination of TCS and GrzB suppressed cell proliferation and induced apoptosis in tumor tissues.

### Induction of apoptosis through the activation of key apoptosis-associated proteins

**Fig. 5:**
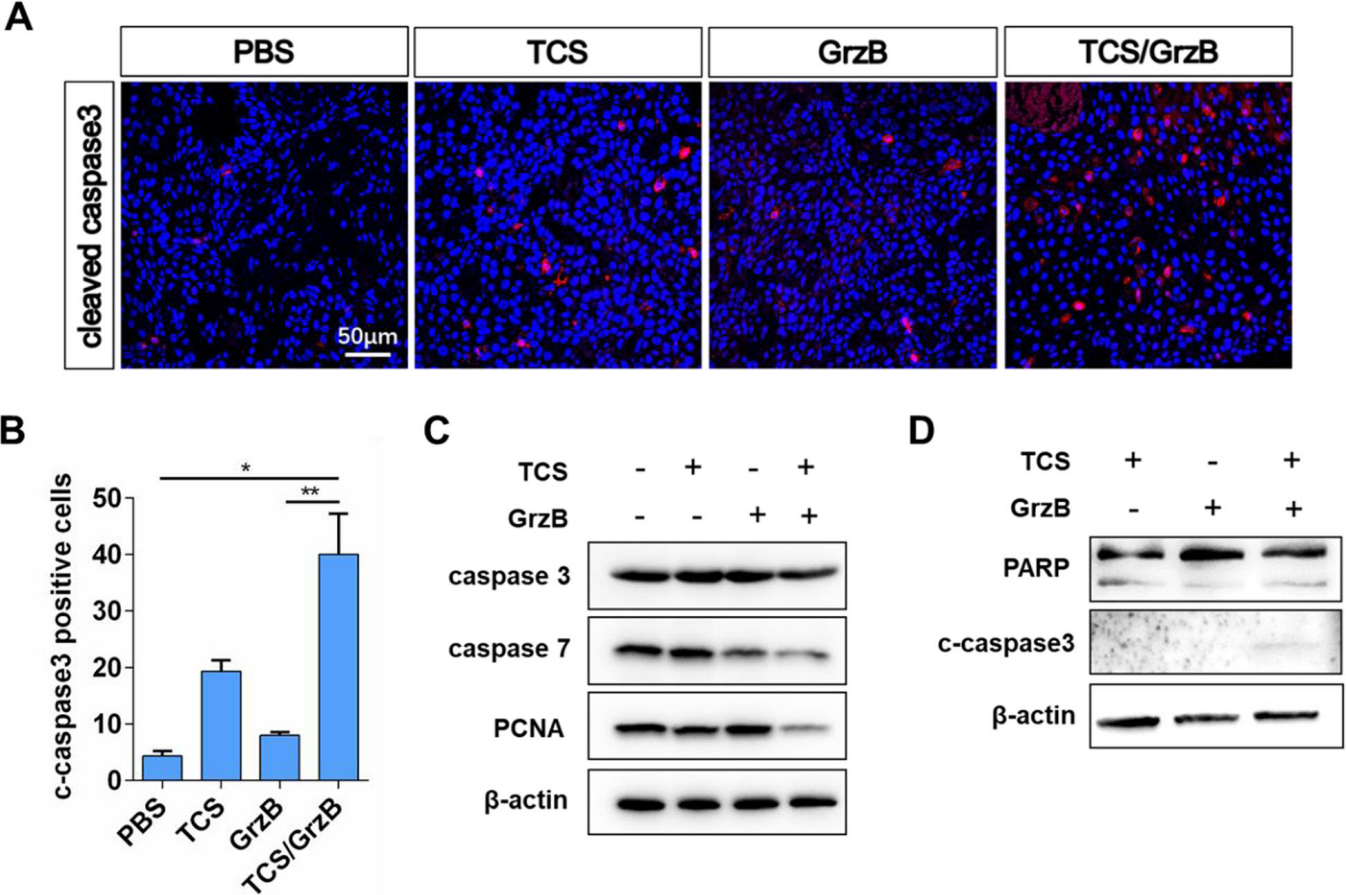
Effect of TCS and GrzB on the expression of apoptosis-associated proteins. **a**, **b**. The positive signals of cleaved caspase3 were greatly reduced in TCS/GrzB group. (cleaved caspase-3: red; DAPI: blue). Bar = 50 μm. **c**, **d**. Western blotting analysis to detect caspase3, caspase7 and PCNA in tumor tissues after various treatments. Data are expressed as mean ± standard error of the mean. *, *p* < 0.05; **, *p* < 0.01

Caspase activation and cleavage of its substrate, PARP, are markers of apoptosis in caspase-mediated apoptosis pathways [24]. We therefore investigated whether SCC25 tumor cell apoptosis was also induced through activation of key apoptosis-associated proteins. Activated caspase-3 and caspase-7 and cleavage of PARP were investigated using immunofluorescence and Western blot analysis. As shown in Fig. 5, a and b, we observed a marked increase in activated caspase-3 in the TCS/GrzB-treated group, whereas activated caspase-3 was rarely seen in the other groups. The key executioners of apoptosis, the precursors of caspase-3 and caspase-7, were decreased in the TCS/GrzB-treated group (Fig. 5, c), and cleaved caspase-3 was only observed in the TCS/GrzB-treated group (Fig. 5, d). However, neither full-length PARP-1 or cleaved PARP-1 levels were significantly changed in tumor tissues. Therefore, the results indicated that TCS induced apoptosis in tumor tissues through the activation of caspase-3 and caspase-7.

**Fig. 6:**
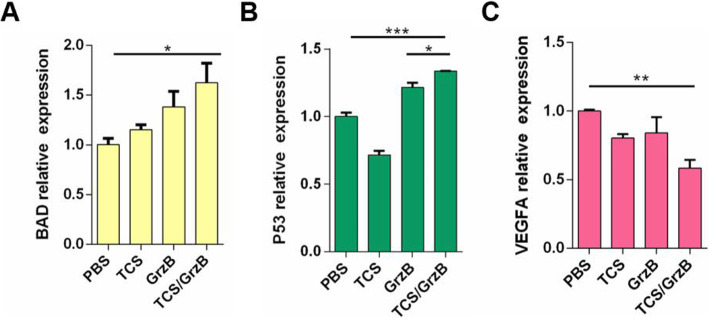
Effect of TCS and GrzB on the expression of tumor-associated mRNA. **a**-**c**. Realtime PCR analysis to detect BAD, p53 and VEGFA in tumor tissues after various treatments. Data are expressed as mean ± standard error of the mean. *, *p* < 0.05; **, *p* < 0.01; ***, *p* < 0.001

BAD, known to be a regulator of programmed cell death, positively regulates cell death by forming heterodimers with BCL-2 and BCL-xL [25]. We found that TCS/GrzB combination treatment markedly enhanced the expression of BAD in tumor tissues compared with its expression in the other treatment groups (Fig. 6, a), suggesting that TCS/GrzB might suppress tumor growth and progression by upregulating BAD. One of the first tumor-suppressor genes to be linked to apoptosis, p53, plays a major role in regulating apoptotic cell death in tumor tissues [26]. We investigated further the possible roles played by p53 in SCC25 cells and found that p53 expression was significantly upregulated in the GrzB and TCS/GrzB groups (Fig. 6, b). This indicated that p53 might play a role in SCC25 tumor apoptosis following TCS/GrzB treatment. Tumor angiogenesis induced by vascular endothelial growth factor A (VEGF-A) plays an important role in tumor growth [27, 28]. VEGF-A expression was examined in tumor tissues of the four treatment groups. It was found that VEGF-A was significantly decreased in the TCS/GrzB treatment group (Fig. 6, c), indicating that TCS cooperates with GrzB to restrain tumor formation by influencing tumor vasculature via the regulation of VEGF-A.

## Discussion

Tongue squamous cell cancer has the highest incidence among all oral cancers, with an approximately 60% 5-year survival rate following comprehensive sequential therapies [4]. Furthermore, TSCC has the highest rates of mortality among head and neck region cancers, constituting a major challenge for head and neck surgeons [3, 6, 7]. The tongue is a highly vascularized and mobile organ, making it impossible to surgically eradicate tongue tumors without tumor cells migrating along circulation [29, 30]*.* In clinical practice, pre- and/or post-surgery chemotherapy or radiotherapy for tongue cancer is always necessary [31–34]. However, the drugs used for TSCC chemotherapy have limited efficacy and unsatisfactory clinical outcomes [35, 36]. In recent years, several new drugs that target tongue cancer cells have been reported [37, 38]. We recently reported that TCS combined with GrzB might be a superior treatment for enhancing the efficacy of liver cancer immunotherapy [17]. In the present study, we investigated the antitumor efficacy of TCS and GrzB combination therapy on TSCC.

TCS is used as an anti-inflammatory agent in traditional Chinese medicine [9, 39]. Recently, TCS has been investigated for its antitumor activity against several types of tumors, based on its ability to induce apoptosis [9, 11, 18, 40–42]. However, TCS was found to exhibit antigenicity and cytotoxicity at high dosages, hindering its clinical applications [43]. In our previous study, we found that low a dose of TCS significantly increased cell surface expression of CI-MPR, a cell-death receptor for GrzB protein transportation during cytotoxic T cell-induced apoptosis [17]. In the present study, we first screened the potential antitumor activity of TCS using two types of SCC cell lines and confirmed that the SCC25 line exhibited greater sensitivity to TCS. TCS also resulted in significantly decreased viability of SCC cells at lower concentrations compared with the reduction in viability obtained using the traditional chemotherapy drug, cisplatin. To investigate the effect of TCS and/or GrzB on TSCC in vivo, a xenograft SCC25 tumor model was established and validated, following a previously established method [37, 38], in which TSCC tumors form 3 days following a subcutaneous injection of a TSCC cell suspension. After 16 days of administration of TCS, GrzB, or a TCS/GrzB combination, the tumors were excised and collected for histology, immunohistology, and immunoblot analyses. It was found that, compared with tumor growth in the control group, TCS or GrzB treatment significantly inhibited tumor growth. TCS combined with GrzB treatment led to a more pronounced inhibition of tumor growth compared with the inhibition resulting from treatment with either drug alone. These results suggest that the TCS and GrzB combination treatment has more potent antitumor activity toward tongue cancers in an in vivo mouse model.

Previous studies have revealed that TCS-induced apoptosis is associated with caspase activation [11, 15, 16]. Caspases are principal effectors that play critical roles in the induction of apoptosis, cleaving targets to execute cell death [44, 45]. Caspase-3 and caspase-7 have long been recognized as the key proteases in the cell demolition processes involved in apoptosis, through the targeting of structural substrates including cell–cell adherence junctions, focal adhesion sites, and nuclear laminins [46]. These two caspase proteases function in a similar way during the execution phase of apoptosis. It has been reported that caspase-3-deficient cells can continue apoptosis if caspase-7 is present [47]. However, apoptosis cannot occur in cells lacking both caspase-3 and caspase-7 [48]. In the present study, cleavage of both caspase-3 and caspase-7 was observed in the TCS and GrzB-treated groups, indicating both caspase-3 and caspase-7 take part in apoptosis.

The development of oral cancer is a complicated process, driven by multiple genes and involving multiple steps. Generally, the hallmarks of cancer comprise six biological processes: sustaining proliferative signaling, evading growth suppressors, resisting cell death, enabling replicative immortality, inducing angiogenesis, and activating invasion and metastasis [49]. To mechanistically explain our observations, we also investigated the effect of TCS treatment on markers of tumor proliferation (Ki67, PCNA). Our present study demonstrated that TCS and GrzB decreased cell proliferation levels. Targeting angiogenesis to prevent tumor progression is a novel direction for cancer therapy [50]. In a previous study, He et al reported that VEGF expression and secretion were significantly decreased in JAR cells following treatment with TCS [51]. Here, we observed a decrease in the expression of angiogenesis marker (VEGF-A) in tumor tissue extracted from the TCS/GrzB treatment group. These results suggest that the VEGF pathway is involved in the anti-angiogenic effects of TCS and GrzB.

## Conclusion

The combination of TCS and GrzB treatment exhibited superior inhibitory effects on SCC25 tumor growth in vivo. TCS and GrzB suppressed SCC cell proliferation by downregulating Ki67, PCNA, VEGF-A, and Bcl2 expression, while accelerating apoptosis via the upregulation of cleaved caspase-3, caspase-7, and BAD activity. The combination of TCS and GrzB could represent a more potent immunotherapeutic protocol for the treatment of oral squamous cell carcinoma, and further investigations are warranted.

## Data Availability

The datasets used and analyzed during the current study are available from the corresponding author on reasonable request.

## Abbreviations

TCS: Trichosanthin
GrzB: Granzyme B
OSCC: Oral squamous cell cancer
TSCC: Tongue squamous cell cancer
CTL: Cytotoxic T lymphocyte
CI-MPR: Cation-independent mannose-6-phosphate receptor
HEPES: Hydroxyethyl piperazine ethane sulfonic acid
IACUC: Institutional animal care and use committee
SPF: Specific pathogen-free
HE: Hematoxylin and eosin
HRP: Horseradish peroxidase
DAPI: 4′,6-diamidino-2-phenylindole
TUNEL: Terminal deoxynucleotidyl transferase-mediated dUTP nick-end labeling
VEGF-A: Vascular endothelial growth factor A
BAD: BCL2-associated agonist of cell death
